# Cell Replacement Therapy for Type 1 Diabetes Patients: Potential Mechanisms Leading to Stem-Cell-Derived Pancreatic β-Cell Loss upon Transplant

**DOI:** 10.3390/cells12050698

**Published:** 2023-02-22

**Authors:** Ali H. Shilleh, Holger A. Russ

**Affiliations:** 1 Barbara Davis Center for Diabetes, School of Medicine, University of Colorado Anschutz Medical Campus, Aurora, CO 80045, USA; 2 Department of Pharmacology and Therapeutics, School of Medicine, University of Florida, Gainesville, FL 32610, USA; 3 Diabetes Institute, School of Medicine, University of Florida, Gainesville, FL 32610, USA

**Keywords:** cell replacement therapy, type 1 diabetes, stem-cell-derived β-like cells, autoimmune diabetes, transplantation, ischemia, transdifferentiation, dedifferentiation, cell death, pancreatic progenitor

## Abstract

Cell replacement therapy using stem-cell-derived insulin-producing β-like cells (sBCs) has been proposed as a practical cure for patients with type one diabetes (T1D). sBCs can correct diabetes in preclinical animal models, demonstrating the promise of this stem cell-based approach. However, in vivo studies have demonstrated that most sBCs, similarly to cadaveric human islets, are lost upon transplantation due to ischemia and other unknown mechanisms. Hence, there is a critical knowledge gap in the current field concerning the fate of sBCs upon engraftment. Here we review, discuss effects, and propose additional potential mechanisms that could contribute toward β-cell loss in vivo. We summarize and highlight some of the literature on phenotypic loss in β-cells under both steady, stressed, and diseased diabetic conditions. Specifically, we focus on β-cell death, dedifferentiation into progenitors, trans-differentiation into other hormone-expressing cells, and/or interconversion into less functional β-cell subtypes as potential mechanisms. While current cell replacement therapy efforts employing sBCs carry great promise as an abundant cell source, addressing the somewhat neglected aspect of β-cell loss in vivo will further accelerate sBC transplantation as a promising therapeutic modality that could significantly enhance the life quality of T1D patients.

## 1. Introduction

The pancreas consists of two main compartments, the exocrine and endocrine tissue, both with distinct functions. Exocrine tissue consists predominantly of acinar cells that release digestive enzymes into the duodenum via a ductal system, making up most of the cell mass found in the organ. The pancreas also contains endocrine cells that are organized together into highly vascularized cell clusters called the islets of Langerhans. Endocrine cells within islets secret hormones that exquisitely regulate and maintain blood sugar levels within a tight physiological range. Representing only about ~1–2% of the organ tissue, the main endocrine cells are insulin-producing β-cells, glucagon-producing α-cells, somatostatin-producing δ-cells, polypeptide-producing PP cells, and ghrelin-producing ε-cells [1]. Out of all endocrine cells, only β-cells express and secrete insulin in response to elevations in blood glucose levels. β-cell dysfunction or loss is key in contributing toward the development of diabetes, and much research has focused on this fascinating cell type.

Diabetes presents as two major subtypes. In both, the inadequate release of insulin results in hyperglycemia that can be life-threatening. The most common diabetes form, type 2 diabetes (T2D), affecting 462 million people globally, is characterized by the insulin resistance of peripheral tissues and (subsequent) β-cell dysfunction, exhaustion, and loss [2]. In type 1 diabetes (T1D), the patient’s own insulin-producing β-cells are specifically destroyed through an autoimmune-mediated attack predominantly of T-cells, resulting in insulin deficiency. Type 1 diabetes (T1D) is a chronic condition that affects 1 in 500 Americans by the age of 15 [3]. Current treatment for both T1D and late-stage T2D consists of injecting endogenous insulin. Exogenous insulin replacement therapy falls short of recapitulating the exact physiological function of a β-cell, and patients are susceptible to acute and long-term complications [4,5,6]. Hypoglycemic conditions, induced by injecting too much insulin, can result in a life-threatening coma and are a constant risk for patients living with T1D and a practical cure that would alleviate the risks and concerns of current insulin therapy is desperately needed. Therefore, research efforts have focused on promoting cell replacement therapy approaches, such as β-cell proliferation and/or neogenesis and islet transplantation, to identify a practical cure for T1D patients. In this review, we will discuss aspects of cell replacement therapy with a focus on current and potential underappreciated challenges associated with stem-cell-derived β-cell transplantation.

## 2. Current and Potential Cell Replacement Strategies for T1D Patients

### Islet Transplantation to Restore β-Cell Mass

A proof of principal for a potential practical cure has been shown with the establishment of the Edmond protocol in 2000. In this protocol, isolated allogenic cadaveric islets are infused in the portal vein of long-standing T1D patients that receive non-steroid immunosuppression [7,8]. Importantly, islet recipients achieve on average ~35 months of insulin independence [9]. Subsequently, islet transplantation was often performed in conjunction with kidney transplantation [10]. However, there are several challenges associated with this procedure that prevent it from becoming widely accessible for patients. A major drawback with islet transplantation is the limited availability of high-purity isolated human cadaveric donor islet material. This is required to restore euglycemia in patients. Typically, each patient receives 10,000 islet equivalents (IEQs) per kilogram of body weight, an amount that usually needs to be extracted from two donor pancreases. In addition, initial clinical trials showed some patients requiring multiple islet infusions throughout the study, further highlighting the need for an abundant source of functional insulin-producing cells. The chronic immune suppression of patients, especially in children and adolescents, is problematic due to long-term complications, including severe and chronic infections and malignancy. In addition, studies have shown that immune suppressive agents impair β-cell function and survival using animal models [11,12]. Indeed, functional cadaveric islet grafts are frequently lost within 2–5 years due to recurring autoimmunity, side effects of immunosuppressants, and other unknown mechanisms [9,13]. The lack of sufficient donor islets has prompted the search for alternative and abundant sources of functional β(-like) cells for replacement therapy purposes, and much progress has been made using different approaches.

Since porcine insulin has been shown to be physiologically well-matched to humans, the xenotransplantation of porcine islets has been considered an effective strategy to provide adequate amounts of islet material to treat T1D patients. However, immunological responses, such as instant blood-mediated inflammatory reaction (IBMIR) [14,15,16], hyperacute, and cellular rejection, remain major hurdles to overcome and improve porcine islet survival [17,18,19,20,21]. Therefore, several strategies have been explored to overcome immune complications in this setting. The development of genetically modified pigs lacking the expression of certain surface proteins that play key roles in immune rejection upon porcine islet transplantation demonstrates promising results in improving porcine islet survival through combating IBMIR and hyperacute rejection. Preclinical studies also revealed that the blockade of co-stimulatory cell surface molecules suppresses T cell activation, hampers cellular rejection, and improves islet survival in vivo. With no evidence of porcine endogenous retrovirus (PERV) transmission, clinical studies of porcine islet xenotransplantation in T1D patients showed initial successes; however, most recipients failed to maintain long-term normal glycemic levels. Encapsulating pig islets has been suggested as effective in reducing xenogeneic immune rejection and prolonging graft survival and was tested in two clinical studies in T1D patients but showed only minimal reduction in their daily insulin needs [22,23,24,25,26].

## 3. Alternative Approaches to Increase Functional β-Cell Mass

Other strategies have aimed at inducing β-cell replication or neogenesis via the transdifferentiation of pancreatic non-β cells to replenish the β-cell mass. Recently, DYRK1A inhibition in conjugation with other pathway manipulations has been shown to be effective in inducing increased β-cell proliferation [27,28,29,30,31,32]. If safe β-cell-specific delivery modalities can be identified, inducing the proliferation of remaining β-cells in diabetic patients might provide a viable therapeutic strategy [33].

Transdifferentiation refers to the change in functional cell phenotype of a differentiated cell into another rather than the differentiation of a less specialized stem cell into a functional cell type. Pancreatic duct ligation (PDL) has been shown to promote β-cell transdifferentiation from ductal, acinar, and alpha cells in mice [34,35,36,37], although some observations could not be repeated in another study [38]. Similarly, the overexpression of *MafA*, *Pdx1*, and *Ngn3* can trigger β-cell transdifferentiation predominantly from mouse acinar cells in vivo [39]. Recently, a group induced alpha cell transdifferentiation into β-cells in vivo by infusing adeno-associated viruses carrying *Pdx1* and *MafA* into the pancreatic duct of NOD mice [40]. However, such experimental strategies, while carrying the potential for endogenous β-cell repopulation in T1D patients, are awaiting translation to human systems and/or clinical settings.

## 4. Stem-Cell-Derived β-Cells as an Abundant Cell Source

**Figure 1:**
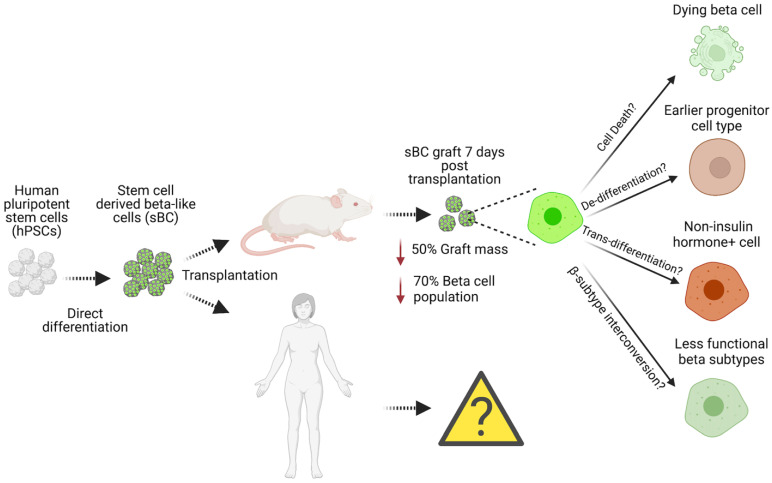
Reported and potential molecular and cellular mechanisms driving human pancreatic β-cell loss upon transplantation.

One attractive approach that has advanced rapidly and shows tremendous potential as an abundant source of functional insulin-producing cells for clinical use is the direct differentiation of human pluripotent stem cells (hPSCs) into stem cell-derived β-like cells (sBCs) [41,42,43,44]. Mouse development studies have identified critical transcription factors and signaling events during pancreas organogenesis, and subsequent work defined the necessary culture conditions to mimic key development stages to direct the differentiation of sBC from pluripotent stem cells [45,46,47,48]. Specifically, several groups focused their efforts on utilizing recombinant proteins and small molecules to generate subsequent cell types resulting in pancreatic cells: definitive endoderm generation [49,50], posterior gut specification [50,51,52,53], formation of pancreatic bipotent progenitors [54,55,56], and endocrine differentiation [41,42,43,50,57]. Although sBCs generated with early protocols were glucose-responsive, cells still displayed features of immature, fetal-like β-cells, and thus performed poorly in dynamic glucose-stimulated insulin secretion (dGSIS) perifusion assays. Several methods, such as the manipulation of key signaling pathways [58,59,60], media composition and in vitro culture extension [61,62], and the use of surface markers and fluorescence-activated cell sorting (FACS) to enrich reaggregated sBCs resulted in a more mature β-cell phenotype that closely resembles primary adult islets [22,44,62,63,64]. Clear criteria defining a mature, functional β-cell that allows distinction from β-cell surrogates has recently been discussed in detail elsewhere [65]. Interestingly, sBC maturation also seems to be accomplished upon transplantation, which in return restored euglycemia and reversed diabetes in preclinical mouse models [41,42,43,66,67]. However, the early events taking place during the immediate engraftment of sBC have not been studied in detail. A considerable body of work has shown that the majority of functional β-cell mass is lost from human islets upon engraftment, suggesting that such drastic effects may also apply to sBCs due to unknown underlying cellular and molecular mechanisms [68]. Potential mechanisms that might occur are: (i) cell death, (ii) dedifferentiation, and (iii) transdifferentiation. Recent work identified distinct human β-cell subpopulations in sBC and human islets in vitro and provided the possibility of (iv) β-subtype interconversion upon engraftment as an additional mechanism. Hence, there is a critical knowledge gap in the research field concerning the fate of sBC upon engraftment. Expanding our knowledge of the contributing mechanisms would expedite our progress in promoting the current approaches of delivering sBC as an effective cell replacement therapy for T1D patients. In addition, sBC cell therapy might represent an attractive treatment modality for T2D patients due to the absence of reoccurring autoimmunity if allogeneic rejection can be avoided in a localized manner. In this review, we will discuss findings and potential mechanisms driving β-cell loss upon engraftment (Figure 1), its implications for cell replacement therapy, and strategies to improve our understanding of the events affecting human β-cells upon transplantation.

## 5. Engrafted β-Cell Loss via Cell Death

Classically, the main mechanism associated with islet cell death in vitro and in vivo is apoptosis. Terminal deoxynucleotidyl transferase dUTP nick end labeling (TUNEL) has shown that a considerable proportion of isolated human islets harvested from donor pancreases are lost in vitro within 5 days of culture due to apoptosis [69]. The high propensity of β-cells to undergo apoptosis within islet preparations might be due to the increased metabolic rate that is not met under cell culture conditions. Later studies determined the caspase cascade as the major intrinsic mediator of apoptosis in cultured islets after being exposed to toxic levels of glucose [70]. Glucotoxicity downregulated BCL-2 (anti-apoptotic protein) expression in isolated islets, which acts as an intrinsic signal to activate caspase-associated apoptosis [71,72]. Similarly, after 24 h of transplantation, TUNEL staining showed that approximately a quarter of all β-cells in human islets engrafted in the kidney capsule of immunodeficient mice are lost due to apoptosis [73]. Overall, although the exact mechanisms contributing to primary β-cell death upon transplantation are poorly understood, the current literature predominantly attributes the observed loss to ischemia and nutrient deprivation. Mediated by endothelial cells, in situ, pancreatic islets are highly vascularized and are under a continuous supply of oxygen and nutrients, ensuring optimal function. However, this supply is lost during the islet isolation process, which involves the use of digestive enzymes and mechanical force to separate the islets from the native organ [74]. Due to loss of blood flow and imperfect culturing conditions, endothelial cells, which are critical in providing cellular matrix proteins that fine-tune the function of β-cells, eventually die in vitro [75]. Since blood flow is abolished after isolation, islets are under an acute nutrient deficiency and exposed to oxidative stress mediated by hypoxia. Isolated islets depend on passive nutrition diffusion to satisfy the activities of the highly metabolic β-cells. Thus, culture conditions are insufficient in supplying uniform O_2_ levels to all β-cells, especially cells located at the core of the islet, negatively effecting β-cell survival in vitro, with necrotic cores present, especially in larger islets due to low oxygen accessibility [76,77,78]. Similarly transplanted human and rodent islets have been shown to have reduced graft oxygen tension in the initial stages of engraftment and to suffer a drastic loss of β-cells in vivo [79,80,81,82,83,84]. Therefore, several in vitro pre-transplant priming methods have been adopted to improve islet survival in transplants, such as oxygenation treatment, culture in hyperoxic conditions, and modulation of seeding density; however, these strategies have failed to be exceedingly successful [85,86,87]. Further mechanistic analysis revealed several signaling pathways, such as anaerobic glycolysis and hypoxia-inducible factor (HIF)-related pathways, to be associated with β-cell survival under hypoxic conditions; however, further investigation is required [84,88,89,90,91,92,93]. Finally, other necrotic-regulated mechanisms such as pyroptosis [94,95], ferroptosis [96,97,98,99,100,101], and necroptosis [102,103,104,105,106,107] have been implicated to contribute toward β-cell death during islet isolation, culture, and transplantation; however, these mechanisms have not been comprehensively elucidated as of yet.

Most in vivo sBC studies have focused on the metabolic action and long-term therapeutic capacity of engrafted, surviving cells using preclinical animals starting at 3–4 weeks post-transplantation when grafts are fully vascularized. However, most studies have largely neglected the early phase of sBC transplantation. In a recent study, sBCs constitutively expressing luciferase were transplanted subcutaneously or under the kidney capsule of immunodeficient mice, and total graft mass was quantified using bioluminescence [68]. As expected, on the day of transplantation, robust expression of luciferase was detected; however, 7 days post-transplant, this expression was significantly reduced in both sites, indicating substantial graft loss. In addition, the hPSC cell line employed also contains a GFP reporter driven by the insulin promoter, allowing quantification of sBCs before and after transplant. Flow cytometry analysis revealed that approximately 70% of sBCs were lost, while the total graft was only 50% reduced within the first 7 days of engraftment, indicating a preferential loss of sBCs compared to other cells present. The main drivers of graft loss are considered to be ischemia-induced hypoxia and nutrition deprivation. Amino acid supplementation and adjusting the physiological oxygen levels to 5% in culture improved sBC graft survival significantly. In situ, pancreatic islets are abundantly vascularized with a continuous supply of oxygen and nutrients; therefore, this study further highlights the importance of vascularization to sBC survival and function in vivo. Pepper and colleagues showed the pre-vascularization of the subcutaneous site followed by the transplantation of pancreatic endoderm (PEC) cells improved stem cell-derived β-cell functionality and survival in vivo, providing further evidence for the notion that appropriate vascularization is critical for β-cell survival and function [108]. Several groups focused on engrafting sBCs that incorporate endothelial cells alone or in combination with mesenchymal cells [109,110,111,112,113]. In a recent elegant study, micro-vessels isolated from adipose tissue have been shown to improve and accelerate the vascularization of sBC grafts, as well as their survival and function in vivo [114]. Finally, using oxygen-generating biomaterials shows promising results to improve islet survival in vivo that could be applied to future sBC engraftments [109,113,115,116,117,118,119]. Altogether, the literature has provided data suggesting hypoxia and nutrient deprivation as two key contributors to sBC graft decline that can be mitigated by providing better engraftment solutions. Understanding what distinguishes sBCs that survive the first week of engraftment from sBCs that are lost during this period could provide additional means to preserve total functional graft mass.

## 6. De-Differentiation upon Transplantation: Do β-Cells Revert to Progenitor/Precursor Cells?

Cell death is the most explored mechanism contributing to immediate β-cell loss upon transplant in vivo, while other potential means resulting in the observed β-cell loss have not been thoroughly investigated. One such mechanism could be β-cell dedifferentiation. Dedifferentiation is loosely defined as the loss of a mature, functional β-cell phenotype and/or the acquisition of progenitor/precursor traits [120,121]. Dedifferentiation of mouse β-cells has been shown by: upregulation in the expression of progenitor genes (e.g., *Foxo1*, *Neurog3*, and *Aldh1a3*), enrichment of disallowed β-cell makers (e.g., *Hk2*, *Ldha*, and *Mct1*) [122,123], mis-localization, loss or reduced expression of key β-cell transcription factors (e.g., *Nkx6.1*, *MafA*, and *Pdx1*), and altered expression of metabolic genes (e.g., *Glut2* and *Gck*) [123].

In vitro and in vivo models of type 2 diabetes (T2D) have suggested oxidative stress, ER stress, and nutritional stress as main contributors to β-cell dedifferentiation. The loss of FOXO1 expression has been suggested as a key trigger of β-cell dedifferentiation. Mice lacking *Foxo1* developed hyperglycemia and β-cell dysfunction [124,125]. A lineage-tracing analysis revealed that *Foxo1*-deficient β-cells dedifferentiated into a progenitor cell population expressing NEUROG3 (a key early endocrine progenitor maker), as well as the early developmental markers OCT4, NANOG, and L-MYC [124]. Another recent study revealed a decline of FOXO1 expression as well as key β-cell markers NKX6.1 and MAFA in db/db mouse islets and human islets isolated from T2D patients compared to controls [126]. Additional analysis revealed an upregulation of the progenitor marker ALDH1A3 [127], specifically in β-cells of T2D patients, providing evidence for dedifferentiation in humans similar to mice [128]. These results were further supported by earlier animal studies using *Foxo1*^-/-^ or db/db deficient mice, in which mouse β-cells similarly showed an upregulation in ALDH1A3 and NEUROG3 with concomitant downregulation of the expression of β-cell markers MAFA and NKX6.1 [127,129,130]. SOX9, a transcription factor critical for pancreas development, has also been suggested as a novel regulator of β-cell dedifferentiation into a developmentally earlier, pancreatic progenitor-like cell type. The von Hippel–Lindau/hypoxia-inducible factor (VHL/HIF) has been implicated previously to regulate cellular responses to hypoxia [131,132]. Hebrok and colleagues have shown that the deletion of *Vhlh* in mice resulted in glucose intolerance, reduced β-cell mass, and decreased the expression of key β-cell markers (*MafA*, *Pdx1*, and *insulin*) [133]. Further protein analysis revealed the loss of *Vhlh* in mice triggered a progenitor program that resulted in the emergence of SOX9-expressing cells. Using mouse and rat insulinoma cell lines, hypoxic oxygen levels triggered similar dedifferentiation programs in cultured β-cells by perturbing the expression of key β-cell genes while increasing *Sox9* expression [63].

Investigating β-cell dedifferentiation in the human setting has been more challenging. A series of early reports suggested that β-cells dedifferentiate upon culturing in vitro, resulting in the derivation of a proliferative cell population but also a significant reduction in insulin expression [134,135,136,137,138]. However, the dedifferentiation hypothesis was contested by others as simply being the result of β-cell death and the expansion of pre-existing mesenchymal cells within cell preparations. Using genetic lineage-tracing analysis on isolated primary human islets employing Cre/lox technology that was previously restricted to transgenic animals provided direct evidence for β-cell dedifferentiation as initially postulated [139]. These results were subsequently confirmed by another group [140]. Subsequent work revealed a critical role in the activation of key developmental pathways during the dedifferentiation process, suggesting potential leverage points to prevent β-cell dedifferentiation [141]. Indeed, the inhibition of signaling pathways induces redifferentiation of expanded, dedifferentiated human β-cells marked by increased insulin and key β-cell marker expression [142,143,144,145,146,147]. Similar experiments using lineage-traced mouse islets demonstrated mouse β-cell dedifferentiation in vitro but a lack of proliferation, unlike human β-cells, providing an example of distinct species differences [148]. Although it is challenging to prove the occurrence of dedifferentiation of human β-cells in situ, recent studies implicated dedifferentiation events. The increased frequency of a chromogranin A-positive hormone-negative (CPHN) population in T1D and T2D isolated human islets sections and decreased β-cell levels have been reported and could represent dedifferentiation, although the authors suggested emerging regeneration as the sources of CPHN cells [149,150,151]. Similarly, higher levels of non-endocrine cells compared to endocrine-expressing cells were detected in sections of human islets transplanted into T1D patients [152]. Taken together, these studies provide strong support for the occurrence of β-cell dedifferentiation in mice and humans and point toward its potential implication in sBC transplantation settings. However, additional studies are needed to provide rigorous evidence for the potential dedifferentiation of human β-cells in different transplantation settings. Taking advantage of genetic lineage tracing or barcoding strategies would likely provide key advances to fill current knowledge gaps.

## 7. Transdifferentiation upon Transplantation: Do β-Cells Convert into Other Hormone-Expressing Cells?

Another potential mode of β-cell loss upon transplantation could be transdifferentiation. Transdifferentiation is referred to as the loss of the β-cell phenotype and acquisition of features of other endocrine hormone-expressing cells. Transdifferentiation can occur via two different mechanisms: a direct shift into displaying characteristics of other hormone-expressing endocrine cells in addition to the loss of β-cell features, or indirectly via dedifferentiating into a precursor/progenitor stage first, followed by the acquisition of other endocrine cell features. All endocrine cells arise from early pancreatic cells marked by PDX1 expression during development [153,154]. Pancreatic lineages become further specified by specific transcription factors, some of which exhibit antagonistic actions at key lineage decisions by inhibiting each other. PTF1α and NKX6.1 are critical in specifying pancreas progenitors further. PTF1α expression gives rise to exocrine tissue [155,156]. In contrast, NKX6.1 expression [157,158] segregates the ductal and endocrine lineages from the exocrine acinar cells during development [36,159,160]. Notch induction of bipotent progenitors marked by PDX1 and NKX6.1 expression triggers endocrine differentiation, giving rise to a transient expression NEUROG3 [161,162,163]. This is followed by endocrine cell specification, which is also governed by key transcription factors: PAX4 expression gives rise to β-and δ-cells [164,165], while *Arx4* is necessary for α-cell development [166,167]. Using knock-out approaches, these studies showed that ARX and PAX4 act antagonistically to give rise to their respective lineage endocrine hormone cell types. These differentiated endocrine hormone cells have been viewed as terminally differentiated cells that acquire their specialized function and lose the ability to proliferate and differentiate into other cell types. However, several mouse studies have shown that endocrine cells can transdifferentiate into other cell types under forced genetic and diabetic stress conditions.

As a proof of principal, β-cell transdifferentiation into α-cells has been initially explored via altering key transcription factors using mouse models. Overexpression of ARX in all endocrine cells showed a drastic reduction in the levels of β- and δ-cells and increased α- and PP cells [168]. Similarly, the overexpression of ARX in all β-cells resulted in transdifferentiation into α- and PP cells. Furthermore, the loss of key β-cell transcription factors also resulted in β- to α-cell transdifferentiation. Supported by lineage-tracing analysis, endocrine precursor and β-cell-specific KO mouse models of *Nkx6.1* revealed a significant reduction in the β-cells and a significant increase in all other hormone-expressing cells (α, δ, PP, ε) [169]. Further studies revealed enrichment in Neurog3 expression in adult islets of cell-type-specific KO *Nkx6.1* mice, suggesting that β-cells are potentially dedifferentiating into a precursor cell type before acquiring a non-β-endocrine cell phenotype [170]. Similarly, the deletion of the tinman domain of *Nkx2.2* in conjugation with lineage analysis resulted in β- to α-cell transdifferentiation and eventually hyperglycemia in young adult mice (3.5 weeks and older) [171,172]. Importantly, in the described studies, no significant changes in the total endocrine cell mass or any polyhormonal cells were found, suggesting two things: the transdifferentiation events included a loss of β-cell phenotype with some aspects of dedifferentiation first, followed by acquiring an α-cell type phenotype thereafter. Other than pancreatic developmental transcription factors, a recent study ablated *Xbp1* in adult mouse islets, a major regulator of the unfolded protein response (UPR) and β-cell function, which resulted in β- to α-cell transdifferentiation and subsequently in hyperglycemia and diabetes. Interestingly, these studies observed an increase in the expression of the progenitor marker *Sox9*, suggesting a dedifferentiating phase before the β- to α-cell transition [173]. In sum, mouse studies revealed conditions of β-cell transdifferentiation; however, findings were based on hormone expression analysis but mostly lacked comprehensive functional assays.

Only limited observations have been reported documenting human β-cell transdifferentiation in vitro and in vivo. Supported by lineage tracing analysis, the reaggregation of islet cells in vitro resulted in the transition of β-cells into α-cells [174]. Moreover, there are several lines of evidence suggesting the occurrence of transdifferentiation of human diabetic islets. IHC analysis of T2D human isolated islets showed higher levels of bi-hormonal cells expressing glucagon and insulin [175,176]. This study also described a cell population that expresses the α-cell hormone glucagon and the β-cell marker NKX6.1 but not insulin and could represent a midway transdifferentiation phenotype between β- and α-cells. Another study showed higher levels of polyhormonal cells in lean-isolated T2D primary islets, further supporting the occurrence of transdifferentiation [149]. However, these studies were performed on isolated human islets in vitro, and therefore one potential explanation for the emergence of bi/polyhormonal cells could be attributed to the isolation process and poor culturing conditions. Moreover, currently, it is unknown if transdifferentiation occurs in T1D islets due to the rarity of such samples and the difficulty in capturing islets at different stages of the disease in situ.

Currently, the field lacks a comprehensive composition analysis of transplanted pancreatic islets in both human and rodent models. In the setting of sBC grafts, scRNA-seq, protein, and functional analyses showed an improvement in sBC maturation and functionality upon engraftment [66,67]. However, it appears that the graft displays more α-cells than β-cells compared to sBCs in vitro that possess more insulin-expressing cells [63]. Interestingly, polyhormonal cells generated as an unwanted byproduct during the differentiation of sBCs seem to resolve in vivo into α-like cells [63]. Polyhormonal cells lack NKX6.1 expression; thus, the transition of these cells into α-like cells upon engraftment might resemble aspects of the transdifferentiation observed in the *Nkx6.1* KO mice [169,170]. In addition, enterochromaffin cells have recently been identified as an unwanted off-target differentiation product [44]. While examples exist that convincingly demonstrate transdifferentiation in animal models, the available human data are limited. To corroborate transdifferentiation phenomena in sBC transplantation settings will require careful additional experimentation. Understanding the underlying molecular mechanisms of transdifferention, as well as dedifferentiation, might allow formulating strategies to preserve a pristine β-cell phenotype upon transplantation.

## 8. β-Cell Subtype Interconversion upon Transplant

While in the past β-cells have been commonly viewed as a rather homogenous population, functionally different β-cells were already described decades ago, and the concept of β-cell heterogeneity and subpopulations has recently received increased attention [177,178,179]. Indeed, β-cells can be subdivided into distinct subpopulations both in mice and humans. Several molecular markers label mature/immature β-cell subpopulations in mouse islets, such as E-cadherin, FLTP and UCN3 [180,181,182]. Recent advances in scRNAseq revealed 3–5 distinct β-cell subpopulations based on differential mRNA transcription in cadaveric human islets [183,184]. Dr. Grompe and associates identified four β-cell subtypes (β1, 2, 3, and 4) in adult human pancreas marked by the surface protein markers CD9 and ST8SIA1 [185]. A sorting strategy using antibodies against CD9 and ST8SIA1 allowed differential gene expression analysis that revealed distinct transcriptional gene profiles, many associated with β-cell functionality. Glucose-stimulated insulin secretion (GSIS) experiments showed that β1 cells, the most abundant subtype in healthy individuals, are also the most functional subpopulation. Interestingly, the distribution of β-cell subpopulations in cadaveric T2D human islets is skewed towards less functional subtypes. Altogether, this study provides a thorough characterization of human β-subtypes in both healthy and diseased islets; however, the distribution of these subtypes in transplant settings has yet to be discovered.

Aside from molecular markers, β-cell subtypes can be categorized based on insulin secretory profiles [177,186,187], functional properties (pacemaker cells such as Hub cells and first responders) [188,189,190,191,192], and other phenotypes. Supplemented with human islet data, animal studies have identified a subpopulation of β-cells that acquire a senescence and senescence-like secretory phenotype (SASP) [193]. Senescence is a state in which cells cease to divide but remain metabolically active with an altered phenotype. Some but not all senescent β-cells exhibit SASP by secreting a mixture of chemokines, cytokines, and ECM molecules, among others. The activation of the DNA damage response (DDR) due to cellular stress has been demonstrated to give rise to senescence and SASP in different cell systems [194,195,196]. Thompson et al. showed SASP-like β-cells, both mouse and human, exist in higher numbers in T1D compared to healthy pancreatic islets. Other than being growth-arrested, SASP cells exhibit non-cell-autonomous activities by secreting factors that affect the viability and function of neighboring (β-) cells and trigger the chemotaxis of immune cells, which leads to the progression of diabetes in T1D mouse models. Strikingly, using senolytic drugs, the clearance of SASP-like β-cells prevents diabetes in a T1D mouse model and preserves β-cell mass and functionality. Interestingly, a similar senescence/SASP β-subpopulation was also identified and characterized in both human and mouse T2D islets. Similarly, clearance of senescent cells in T2D via senolysis improved glucose levels and β-cell function and identity [197]. Altogether, these findings highlight the consequence of SASP cells on disease development and progression, and it will be vital to determine if senescence and SASP occur in sBCs in vitro and in vivo.

Heterogeneity in sBC clusters is less investigated, but multiple scRNA data sets have been published showing distinct subpopulations. Veres et al. performed the first comprehensive scRNA-seq on stem-cell-derived islets at various stages of the differentiation protocol and revealed an endocrine hormone-expressing cell population consisting of β-, α- and polyhormonal cells, an endocrine non-hormone^+^ population, and a previously unreported enterochromaffin population [44]. However, no detailed analysis focused on the sBC subpopulation was performed. These results were subsequently supported by two other groups identifying similar populations with different cell distributions [44,66,67]. Recently, we adopted a more specific approach by performing scRNA-seq analysis on sorted sBCs and identified 7 β-cell subtypes based on an unsupervised cluster analysis. These data revealed a mature β-cell subpopulation marked by the expression of surface ENTPD3 (also known as NTPDase3) protein. The sorting of ENTPD3^+^ sBC followed by reaggregation and functional evaluation using dynamic GSIS revealed secretion patterns similar to primary human islets, while ENTPD3^−^ sBC did not, providing the first evidence for defined heterogeneity within sBCs [62]. Interestingly, these data also revealed other β-subtypes, such as polyhormonal sBC, a proliferative cell sBC population, IGF2^+^ sBCs, and a distinct CD9-labeled sBC population, suggesting that experiments to further stratify sBCs in vitro and in vivo are warranted. Such studies will cumulatively contribute toward optimizing sBC grafts’ functionality in vivo, further promoting cell therapy as the most practical treatment for diabetic patients.

## 9. Conclusions

The lack of donor islets has prompted us and others to generate sBCs as an abundant source of human functional human β-cells. Preclinical studies demonstrated the ability of sBCs to function in vivo and restore euglycemia in diabetic mouse models. However, in vivo studies have also demonstrated that the majority of sBCs, similarly to cadaveric human pancreatic islets, are lost upon transplantation, but the underlying mechanisms remain largely unknown. Currently, there are multiple ongoing clinical trials in T1D patients with some promising results, further promoting stem cell-derived pancreatic progenitors and sBCs as an attractive therapeutic approach for diabetes treatment. Current literature demonstrates that considerable sBC loss occurs upon transplant into preclinical animal models, suggesting this likely happens in humans as well. Understanding the underlying mechanisms resulting in β-cell loss will allow the formulation of strategies to counteract these undesired effects, thereby providing improvements to current cell replacement efforts. Ultimately, improving β-cell survival and function will reduce the graft size needed to restore euglycemia in patients and thus also reduce the cost of this therapy. Currently, β-cell death is largely perceived as the main contributor to β-cell graft mass loss upon transplant. However, we argue that this basic view might be an oversimplified representation of what actually might occur to β-cells upon transplant. Indeed, recent work has highlighted underappreciated aspects of β-cell biology, most notably the plasticity displayed by β-cells under certain experimental and disease conditions, as well as β-cell heterogeneity. We hypothesize here that β-cell mass loss upon engraftment likely constitutes a mélange of different phenomena occurring in parallel. It will require focused experimental approaches that allow accurate cell fate tracking in vivo to determine if other mechanisms in addition to β-cell death contribute significantly to graft loss. The notion that sBCs could transdifferentiate into other hormone-expressing cells might be exploited for improved β-cell function. Other islet cell types have been shown to be critical for β-cell function; thus, generating stem-cell-derived islet-like structures upon transplant might be beneficial. However, sBCs dedifferentiate into a less differentiated, potentially proliferative state, which could require additional (long-term) safety considerations. Obtaining detailed molecular and cellular insights into potential mechanisms will allow the development of preventative strategies. We envision such efforts to be similar to current research efforts aimed at reducing the well-recognized problem posed by ischemia-induced graft loss. Indeed, many research groups use innovative strategies, most notably in the bioengineering space, to provide adequate oxygen and nutrient supply to islets and/or β-cells immediately after transplant to counteract ischemia-induced detrimental effects. Another important question relates to the presence and/ or formation of distinct β-cell subpopulations upon transplant. Indeed, we and others have shown that β-cell subpopulations exhibit differential functions. Obviously, providing the most functional sBC to patients in the cell replacement setting is highly desired over subpar phenotypes. However, it is tantalizing to speculate how different β-cell subpopulations might interact with the immune system and if an immune-privileged population can be identified and exploited for replacement therapy. Reduced immunogenicity of a small β-cell subpopulation has indeed been proposed in autoimmune animal models previously. Furthering our understanding of human β-cell biology in the setting of sBC transplantation might also provide interesting insights that could apply to autoimmune mechanisms in patients and provide novel model systems to investigate them. Overall, understanding the exact mechanisms contributing to sBC loss upon transplant will allow the development of informed strategies to prevent sBC loss and might allow the delivery of greater numbers of sBC with superior function. Such second-generation cell replacement therapy approaches would effectively provide distinct advantages, such as the need for reduced graft mass to be transplanted. Overall, advances in stem cell-based cell replacement therapy for diabetic patients have been impressive within the last few years, and we anticipate that progress will further accelerate to allow this curative approach to reach a wide patient population in the near future.

## Data Availability

Not applicable.
